# New Appliances and Things Medical

**Published:** 1908-11-14

**Authors:** 


### NEW APPLIANCES AND THINGS MEDICAL.

[We shall be glad to receive at our Office, 28 & 29 Southampton Street, Strand, London, W.C., from the manufacturers, specimens of all new preparations and appliances which may be brought out from time to time.]

### ROBINSON'S BARLEY AND GROATS.

We have lately received samples of Robinson's Patent Barley and Robinson's Patent Groats, manufactured by Messrs. Keen, Robinson and Co., Ltd. Both preparations have stood the test of many years' medical and domestic experience, and have an established reputation. We find, after trial, that both the barley and the groats are of the same high quality as heretofore. The former remains unrivalled for the preparation of barley-water suitable for infants and invalids; and the latter for the preparation of nutritious gruel.

### TAMAR INDIEN GRILLON.

The manufacturers of this long established and well tried remedy for constipation, Messrs. Guérin and Co., of 67 Southwark Bridge Road, S.E., have forwarded us a sample of their preparation, which we have tested by personal experiment. We are prepared to testify to the efficiency of the chocolate-coated lozenge, in which the active principle is enveloped by a layer of that sweetmeat, and to assure those of our readers who have been familiar with this preparation in the past that the standard of its excellency has in no way deteriorated. The sample is accompanied by a pamphlet explaining the manufacturer's views of the action of and indications for the use of Tamar Indien; while we are not prepared to endorse everything which this leaflet contains, it is distinctly very far above the usual level of such compilations. After all, the best advertisement of any commercial article is the satisfaction of purchasers, and Messrs. Guérin are not likely to lack that while they maintain the past and present standard of manufacture of Tamar Indien Grillon.

